# Carbonate Acidizing: A New Insight into Wormhole Propagation under Low Flow Rates

**DOI:** 10.1021/acsomega.5c05664

**Published:** 2025-11-07

**Authors:** Jair R. Neyra, Daniel N. N. da Silva, Cláudio R. S. Lucas, Rafael J. Leitão, Flávio B. da Cruz, Sérgio T. de Camargo Júnior, Mateus P. Schwalbert, Pedro T. P. Aum

**Affiliations:** † Petroleum Science and Engineering Laboratory (LCPETRO), 37871 Federal University of Pará (UFPA), Salinópolis, Pará 68721-000, Brazil; ‡ Brazilian Synchrotron Light Laboratory, Brazilian Center for Research in Energy and Materials, Campinas, São Paulo 13084-971, Brazil; ¶ Petrobras Research Center (CENPES), 42506 Petrobras, Rio de Janeiro, Rio de Janeiro 20031-912, Brazil

## Abstract

Wormholes are channels formed by the dissolution of carbonate rock due to interaction with acid solutions. These wormholes create pathways for fluid flow, playing a crucial role in applications such as production enhancement in petroleum engineering and geological CO_2_ storage. However, the formation of wormholes at low flow rates is not well-explored. This gap is important because low injection rates frequently occur in field operations due to either reservoir constraints or operational limitations. Most models fail to accurately describe wormhole formation at low flow rates in systems with highly concentrated acids and high dissolution power, and experimental data remain scarce. In this study, acidizing experiments were conducted at low flow rates to investigate the pore volume to breakthrough (PVBt) and wormhole morphology behavior. Due to minimal pressure drop under these conditions, partial acidizing was systematically performed. Breakthrough was evaluated by analyzing micro-CT images and measuring the distance traveled by the wormhole in the sample to obtain PVBt estimates. The results suggest that the traditional models tend to overestimate PVBt values at low flow rates, leading to wormhole breakthrough occurring earlier than anticipated. A trend in the PVBt curve was detected at lower flow rates, stabilizing at a stoichiometrically derived limit rather than the indefinite increase predicted by earlier models. The findings of this study contribute to a deeper understanding of reactive flow at low flow rates, improving the accuracy of current models.

## Graphical abstract

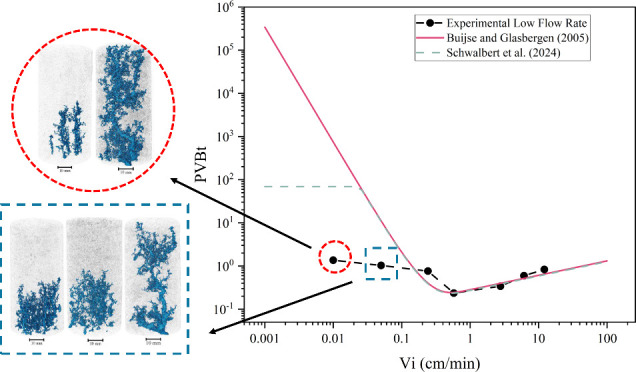

## Introduction

Wormholes are dissolution patterns that form in carbonate rocks as a result of reactive flow, governed primarily by transport dynamics in porous media. These dynamics involve acid–rock reactivity, acid diffusivity, advective transport, and properties of the rock matrix—such as petrophysical characteristics, heterogeneity, and other geological features. In petroleum engineering, acidizing is a well-stimulation technique designed to enhance connectivity between the wellbore and the reservoir.^1,2^ It consists of injecting an acid solution into the target formation at pressures below its fracturing threshold, dissolving minerals along their path and propagating through the porous medium.^3–7^ The wormholes formed are characterized by high-permeability pathways that enhance connectivity between the well and the reservoir.^8^

Optimal wormhole formation results in a single narrow channel that minimizes acid consumption and promotes deeper reservoir penetration. As such, acid efficiency and wormhole morphology are key outcomes with direct implications for field applications.^9–12^

Laboratory experiments are crucial for understanding the propagation of wormholes in carbonate acidizing. Significant advances have been made in the literature on reactive flow.^13–17^ One of the most established parameters for evaluating carbonate acidizing at the laboratory scale is the PVBt (Pore Volume to Breakthrough), which represents the volume of acid required to achieve breakthrough, defined as the point when the dissolution channel reaches the outlet of the core sample, normalized by the rock’s pore volume.^18^ Numerous experiments are conducted to construct the PVBt curve across a range of acid injection rates.^19^

The optimal PVBt is identified as the minimum point on the PVBt curve, indicating the conditions under which acid consumption is minimized for breakthrough. At this point, acid use achieves the most efficient channel formation, producing a narrow, minimally branched wormhole. Extrapolated to field scale, this suggests a wormhole that penetrates maximally into the formation while maintaining minimal acid consumption, a condition desirable for enhancing well productivity or injectivity with optimized resource use.^20^

At laboratory scale, different wormhole patterns are observed. Figure 1 shows a PVBt curve along with these distinct wormhole patterns. Five primary dissolution patterns are highlighted: (1) facedissolution; (2) conical wormhole; (3) dominant wormhole; (4) ramified wormhole; and (5) uniform dissolution.

**Figure 1:**
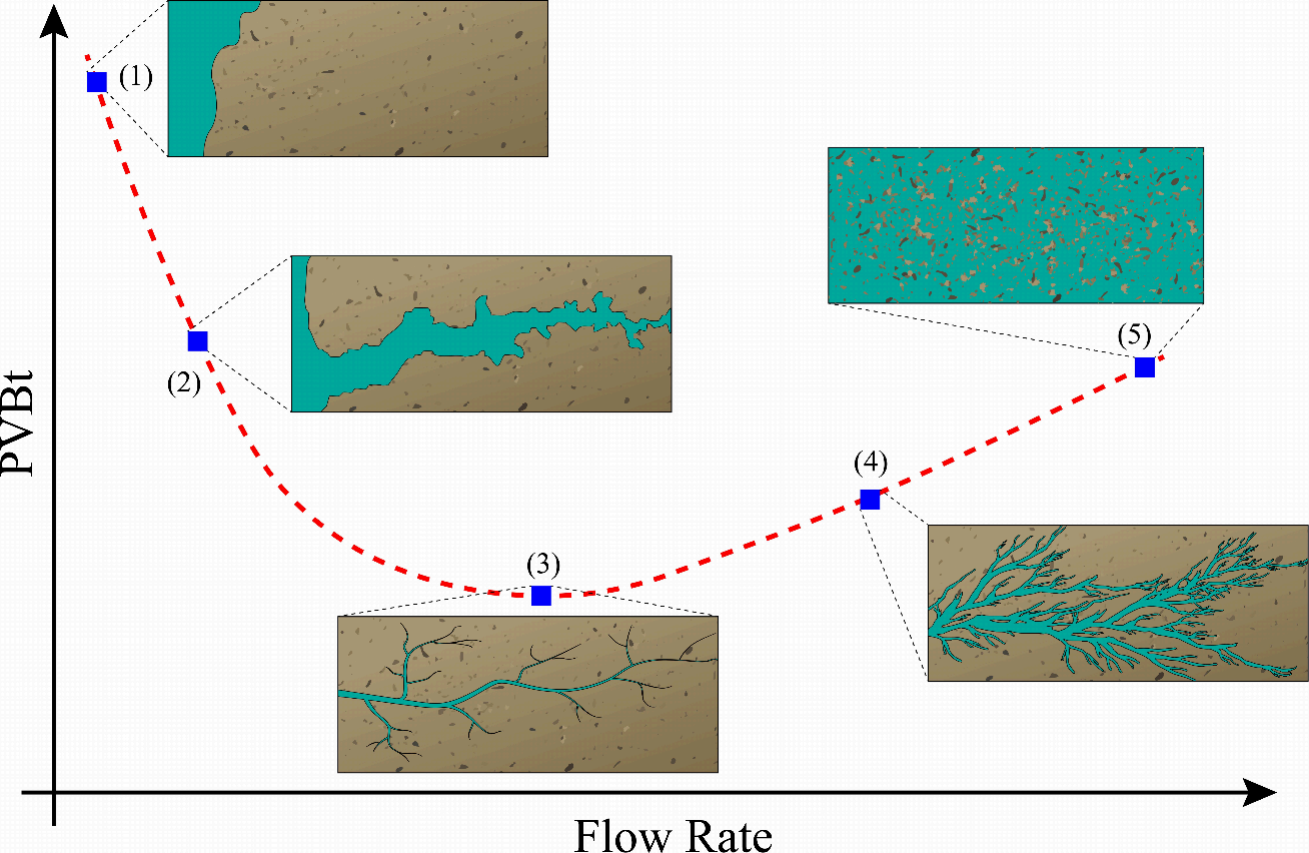
PVBt curve with the corresponding dissolution patterns. The PVBt values along the curve represent the injection flow rates applied during the experiment. Each dissolution pattern is illustrated at a specific point on the curve.

Facedissolution is characterized by a low-rate injection, where advective transport is minimal and the reaction is intense. Increasing the injection rate results in the formation of a conical wormhole, where advective transport rises but the dissolution becomes more pronounced, leading to the creation of a conical shape. The dominant wormhole is the target in these experiments, where only the main channel is created, resulting in reduced acid consumption. This represents an equilibrium between advective transport and reaction. With further increases in injection rate, branches are formed along the main channel, leading to the pattern known as a ramified wormhole. At high flow rates, acid consumption and advancement occur uniformly.^21^

Only a limited number of studies in the literature have reported acidizing experiments at low flow rates. Among those available, when the acid injection rate is below 0.1 cm/min in interstitial velocity, the reported PVBt values are consistently lower than 10, as illustrated in Figure 2. The figure compiles data from different studies and classifies them by acid type:organic, inorganic, and chelating acids. It highlights that lower PVBt values are more commonly associated with high-reactivity acids, such as concentrated HCl. In contrast, higher PVBt values are observed in systems using lower-reactivity acids like chelating agents or organic acids.^2,21–23^ Despite this, most models still overpredict PVBt for highly reactive systems at these low interstitial velocities, indicating a gap between numerical predictions and experimental behavior.

**Figure 2:**
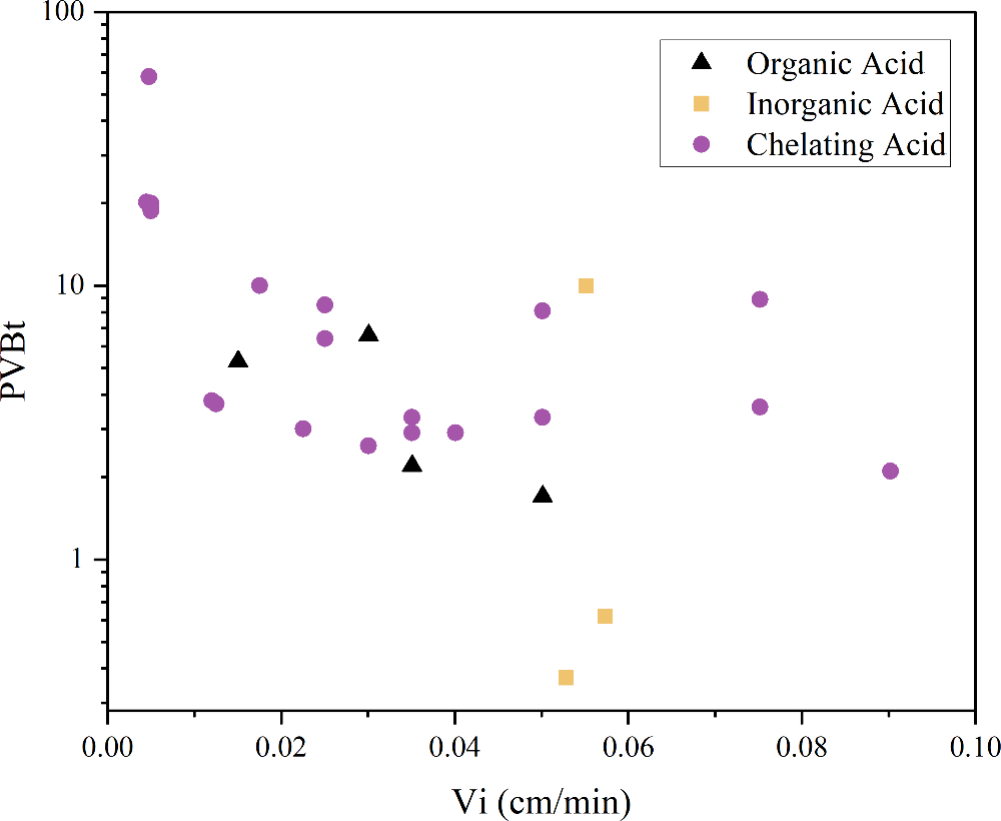
PVBt values obtained from coreflooding experiments conducted at interstitial velocities below 0.5 cm/min, as reported in the literature.^2,21–23^

Various PVBt prediction models, particularly semiempirical ones, have been formulated offering faster results, but often failing to fully capture the physical phenomena. On the other hand, numerical models are more comprehensive but require significant computational time to produce results.

Buijse and Glasbergen^24^ developed a semiempirical model to characterize wormhole propagation, integrating both physical and chemical aspects of the process, calibrated through coreflooding experiments. This wormhole propagation model has been embedded in field simulators and has shown reliability in various applications. However, due to a scarcity of acidizing experiments conducted with high HCl concentrations at low flow rates, the model falls short in accurately capturing the PVBt under these specific conditions.

Similarly, Ali and Ziauddin^25^ developed a mechanistic model, calibrated through reactive flow coreflooding experiments. This model was designed to be straightforward and computationally efficient for predictive purposes, incorporating a wide range of experiments that varied in sample dimensions, rock types, and commonly used acids. The model’s outcomes were satisfactory. However, like the previous model, it was limited by the scarcity of experimental data at low flow rates, particularly with high HCl concentrations. This limitation affects the model’s ability to accurately capture PVBt behavior under these conditions, reducing its predictive accuracy for applications where low flow rates are prevalent.

Schwalbert et al.^26^ developed a new model to conduct numerical simulations using CFD (Computational Fluid Dynamics) to evaluate the effect of acid concentration in scenarios where it is necessary to apply the technique intended to kill a blowout by communicating a relief well to a blowout well through channels formed in the rock by acid dissolution (acid kill). An interesting parameter introduced by the authors is the stoichiometric maximum acid volume required to dissolve the entire rock, providing an asymptotic behavior at low flow rates and correlating it with the concentration of the acid used.

However, PVBt values still reach magnitudes on the order of 10^2^ at high HCl concentrations and 10^3^ at low concentrations. Coreflooding experiments may be required to validate or adjust this trend, thereby enhancing the model’s predictive accuracy at low flow rates.

Experimental studies have used the Buijse and Glasbergen model to fit PVBt results under *CO*_2_ injection conditions. Almalichy et al.^27^ conducted experiments involving *CO*_2_ injection to determine the optimal injection point in carbonate samples, but obtained overestimated PVBt values at low flow rates. Mirzayev et al.^28^ performed experiments with *CO*_2_-saturated brine flow in Indiana limestone samples to assess the geomechanical impact of porous media dissolution. The authors reported that the Buijse and Glasbergen model did not provide good fits at low flow rates, where it also overestimated the PVBt values in this regime.

In this context, the primary objective of this study is to investigate acid consumption and wormhole propagation through experiments conducted at low flow rates. A key challenge in this analysis was determining the PVBt, which is typically observed when the pressure drop approaches zero. However, at very low flow rates, the pressure drop is negligible, close to zero during all the acid injection, making it difficult to determine PVBt using standard methods. To overcome this limitation, a novel approach for coreflooding experiments at low flow rates was proposed.

The experimental strategy consisted of injecting acid at a constant flow rate for a pore volume lower than the expected PVBt, followed by micro-CT analysis of the wormhole pattern and tip advancement into the rock.

Based on the micro-CT images, the PVBt was estimated for each experiment, allowing its determination without reliance on pressure-drop measurement.

The results from these laboratory experiments can contribute to refining the model proposed by Buijse and Glasbergen,^24^ improving its predictive accuracy for PVBt values at low flow rates. A more precise prediction of PVBt under such conditions increases the reliability of simulations for scenarios involving lower injection rates in field applications, enabling operations to be better tailored to reservoir properties. This is particularly relevant for reservoirs such as the Brazilian presalt, where operational constraints associated with deep or low-permeability formations may require, at the beginning of acid treatment, careful control of injection rate and pressure to ensure safe and efficient stimulation.

## Material and Methods

The methodology consists of the following steps: (1) selection of Indiana Limestone outcrop cores, (2) petrophysical measurements of porosity and permeability using a gas porosimeter and gas permeameter, respectively, (3) imaging of the dry cores using micro-CT to visualize their characteristics prior to the reactive flow, (4) saturation of the cores with water, (5) coreflooding experiments, where the rock is subjected to water flow followed by acid injection for a predetermined duration, and (6) visualization of the wormhole formed during acidizing using micro-CT imaging. These steps were systematically carried out to ensure accuracy and reliability of the experimental results. Each step is described in detail in the following sections.

### Preparation of Chemicals and Core Samples

To prepare the acidic solution, ultrapure water and a 36.46% weight hydrochloric acid (HCl) solution with a density of 1.181 g/mL at 25 °C, obtained from Dinâmica Química Contemporânea Ltda, Brazil, were utilized. Additionally, a corrosion inhibitor labeled as 1791/A from Agena Resinas e Colas Ltda, Brazil, was employed. The HCl concentration used was 4.4 M (15 wt%.), achieved through dilution of the concentrated acid solution in ultrapure water. The corrosion inhibitor was introduced at a concentration of 1% (by volume). This type of acid at this concentration was chosen because it is one of the most commonly used in the oilfield industry, attributed to its efficiency in dissolving carbonate rocks and its low cost.^19,29–31^

Eight core samples of Indiana Limestone carbonate outcrops were obtained from Kocurek Industries, Inc., US. Each core had a length of 3 in. and a diameter of 1.5 in., with an average porosity of 18% and a mean permeability of 124 mD. Indiana Limestone is frequently selected for acidizing studies owing to its predominantly calcitic composition (*CaCO*_3_), which accounts for roughly 99%, offering significant mineralogical uniformity.^32^

The core plugs were petrophysically characterized to determine porosity and permeability. Porosity was measured using a gas porosimeter (DCI, USA) based on Boyle’s Law, while permeability was determined with a steady-state gas permeameter (DCI, USA) equipped with Klinkenberg correction to obtain absolute permeability. For each core, three measurements were performed, and the reported values represent the averages. Core saturation was conducted using an automated saturator (DCI, USA), which applied vacuum followed by pressurization with ultrapure water at 2000 psi for 12 h.

### Coreflooding Experiments

The coreflooding system (JBV-USITEC, Brazil), a physical reservoir simulator, was used to conduct reactive flow experiments in porous media. All wetted components were made of Hastelloy to ensure resistance to corrosion. The experimental setup, shown in Figure 3, consisted of pumps, fluid accumulators, pressure transducers, a core holder, a fluid collector, and backpressure regulation.

**Figure 3:**
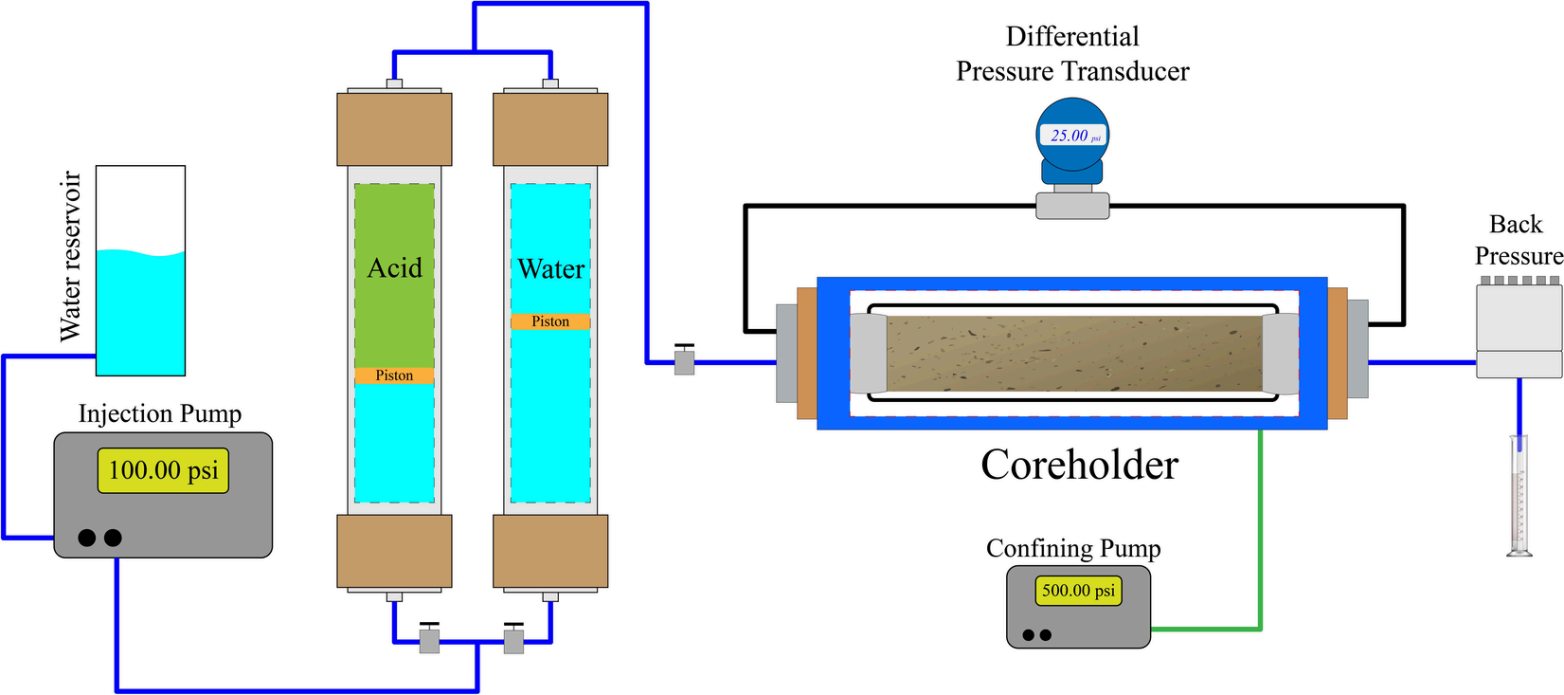
Schematic of the physical reservoir simulator, commonly referred to as a coreflooding system. This equipment enables fluid flow experiments to be performed on rock samples.

To simulate pore pressure and prevent gas formation from acid-rock reactions, a backpressure of 1100 psi was maintained to keep *CO*_2_ in solution. The heating system controlled the temperature of the core holder and fluid accumulators throughout the experiments.

To evaluate PVBt at low flow rates, we adapted the standard acidizing experimental procedure.^2,13,33,34^ Within the flow rate range of 0.02–0.1 mL/min and corresponding interstitial velocities of 0.00947–0.0509 cm/min investigated in this study, no significant differential pressure was observed, which is typically used to indicate breakthrough as the pressure drop approaches zero. To address this limitation, predefined acid injection times were established for each flow rate. After injection, wormhole length was measured using micro-CT imaging, and the pore volume to breakthrough (PVBt) was calculated up to the wormhole tip, as described by eq 1.

1 $${\mathrm{PVBt}}^{*}=\frac{V_{\mathrm{acid‐inject}}\left(t\right)}{{\mathrm{PV}}_{\mathrm{partial}}}=\frac{V_{\mathrm{acid‐inject}}\left(t\right)}{\frac{\pi d^{2}}{4}L_{\mathrm{wormhole‐length}}\phi }$$

This modified approach, referred to as partial PVBt (PVBt*), incorporated as key parameters: the wormhole length measured from micro-CT (*L*_wormhole‑length_), the partial pore volume considering the pore volume of the rock up to the wormhole length (PV_partial_), rock porosity (ϕ), and the volume of acid injected (*V*_acid‑inject_) over time (t).

By varying the injection duration and tracking wormhole propagation, the behavior of reactive flow was analyzed at distinct time intervals. The experiments were carried out in three phases: (1) at a flow rate of 0.1 mL/min for 0.54, 0.72, and 0.81 PV_injected_ (pore volume of acid injected) at 45 °C; (2) at a flow rate of 0.02 mL/min for 0.77, 0.8, and 1.15 PV_injected_ at 45 °C; and (3) at a flow rate of 0.02 mL/min for around 0.8 PV_injected_ at varying temperatures of 25 °C, 45 °Cand 65 °C This setup allowed for the analysis of both flow rate and temperature effects on dissolution and wormhole propagation at low injection rates. The adapted coreflooding sequence for low-flow rates was as follows:

1. Water was injected at three different flow rates to determine the core sample’s absolute permeability. Following this, the flow rate was adjusted to the predetermined value for the acid injection.
2. After water injection stabilized, the flow was switched to acid injection for the predetermined time using a set of valves.
3. Acid injection was stopped, followed by water injection to neutralize the reaction in the rock and clean any acid trace.
4. With the experiment completed, the core was removed from the equipment, placed in an oven at 60 °C to dry, and then sent to micro-CT to visualize the wormhole formed during the experiment.

An additional consideration in this study was the behavior of the rubber sleeve in the core holder. Careful monitoring of the confining and inlet pressures was necessary, as dissolution at the rock face can weaken the rubber, increasing the risk of rupture under the experimental conditions.

### Micro-CT Images

Micro-CT imaging was conducted to visualize the wormholes and the entire dissolution region affected during the experiment. The X-ray computed microtomography (micro-CT) images were acquired using the Phoenix v| tome| x| s 240, and the 3D reconstructions were processed with the Datos| x reconstruction software. Image processing and result generation were performed using Avizo software (version 2024.2) from Thermo Fisher Scientific.

For imaging the cores, a multiscan mode was used with three scans per sample, employing a voltage of 140 kV, current of 160 μA, exposure time of 200 ms, 2500 images over 360°, and a resolution of 22 μm. During reconstruction, several functions were applied to enhance contrast between the rock matrix and pores and to reduce noise, including slice alignment, beam hardening correction, ring artifact reduction, and a mathematical edge-enhancement filter.

To improve segmentation, cuts were made at the top and bottom of the core samples. The samples were segmented into the regions where dissolution occurred and the remaining rock matrix. A 3D view of the complete dissolution and a slice near the base of the sample were obtained, allowing examination of the wormhole structure and the extent of face dissolution. From the complete wormhole and face dissolution, the volume of each component and the length up to the wormhole tip were calculated. Due to the high number of branches, an additional segmentation step was performed to remove surrounding minor structures, improving the visualization of the main channels.

## Results and Discussion

Table 1 shows the partial PVBt* and estimated wormhole length, as well as the petrophysical properties and operational parameters for each experiment. The injected acid volumes were adjusted to account for dead volumes in the injection lines, therefore the PVBt* calculation considers the effective volume of acid injected into each sample.

**Table 1:** Experimental Parameters and Main Results for PVBt* and Wormhole Length

Sample	Flow rate (mL/min)	Injection time (h)	*T* (°C)	Interstitial velocity (cm/min)	Porosity (%)	Wormhole length (cm)	Volume of acid effective injected (mL)	PVBt*
IL_1	0.1	1	45	0.0509	17.22	3.892	7.8462	1.02
IL_2	0.1	1.5	45	0.0490	17.91	4.257	10.84937	1.24
IL_3	0.1	2	45	0.0489	17.94	7.25	12.09933	0.81
IL_4	0.02	8	45	0.00996	17.62	4.607	11.4135	1.23
IL_5	0.02	10	45	0.00909	19.30	3.714	13.8195	1.69
IL_6	0.02	12	45	0.01053	16.66	7.36	16.20778	1.15
IL_7	0.02	10	25	0.00912	19.24	6.819	13.8125	0.92
IL_8	0.02	10	65	0.00947	18.53	6.561	13.70158	0.98

Breakthrough occurred in samples IL_3 and IL_6, however, the exact moment could not be determined due to the low signal in the differential pressure. To calculate the PVBt for these experiments, the total amount of acid injected was divided by the pore volume of the rock. This approach may result in a slightly higher PVBt. The wormhole length for these samples is equal to the total core length because breakthrough occurred.

### PVBt Values at Low Flow Rate

The PVBt curve for HCl 15 wt % (w/w) at 45 °C in Indiana Limestone is presented in Figure 4. The black curve with circle symbols represents the PVBt under normal interstitial velocity conditions, where breakthrough was identified as in a typical experiment. The two points on the black curve, at 0.05 and 0.01 cm/min, correspond to the average of three partial PVBt experiments. For comparison, the individual results are also shown as a green triangle (0.1 mL/min) and pink squares (0.02 mL/min), as previously explained.

**Figure 4:**
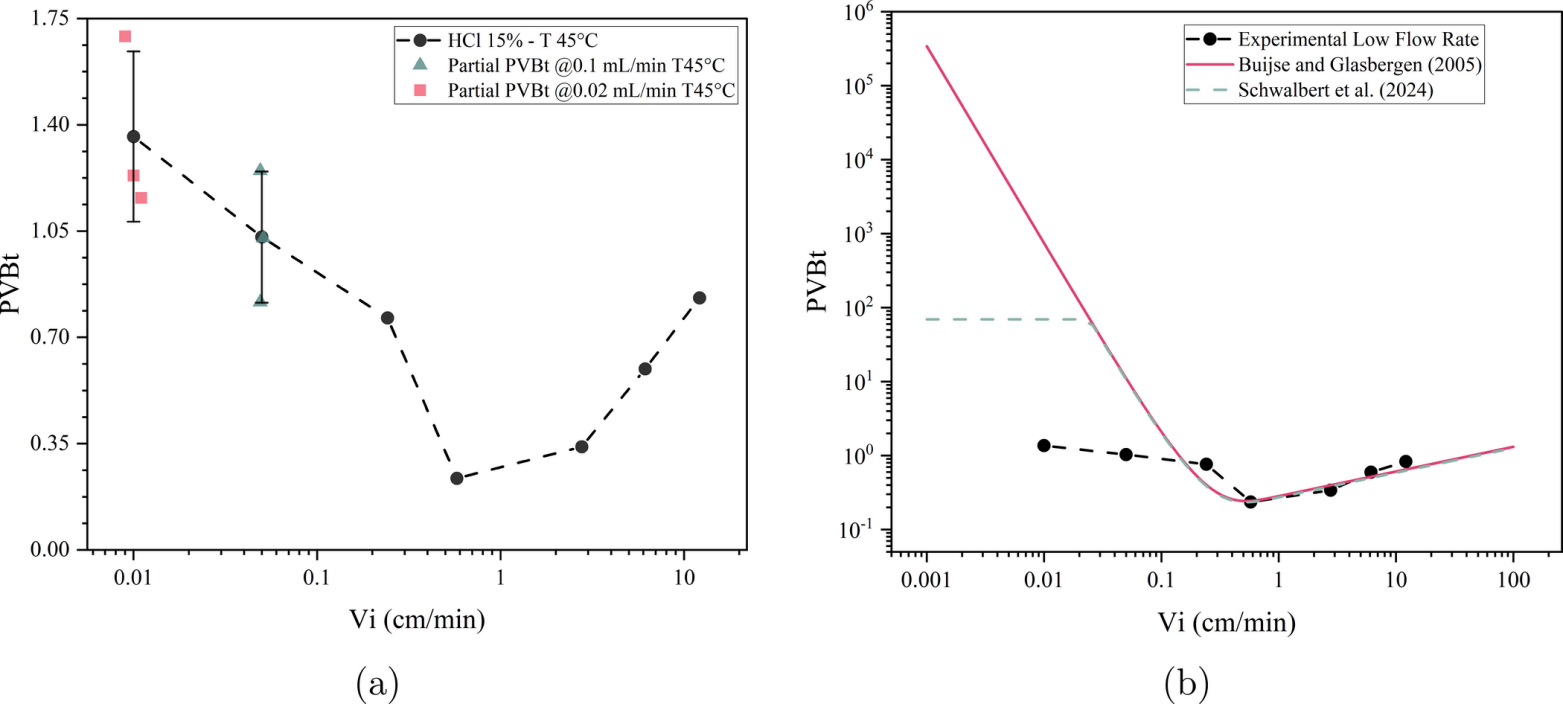
(a) PVBt curve with partial experiments for 15 wt% HCl in Indiana Limestone at 45 °C. (b) The PVBt curve is compared with the Buijse and Glasbergen^24^ model and the newly adjusted numerical model for low interstitial velocities from Schwalbert et al.^26^

Figure 4a shows that the median PVBt values were 1.02 and 1.36 at flow rates of 0.1 and 0.02 mL/min, respectively. However, some variability was observed among the replicates, with maximum values reaching 1.24 and 1.69 for the same flow rates.

A comparison of the PVBt curve behavior from numerical simulation, a semiempirical model, and the experimental results is presented in Figure 4b. The model proposed by Buijse and Glasbergen^24^ is effective at values near the optimal point; however, at lower velocities, the PVBt values are overestimated. For the same low flow rate conditions as in the experiments, Buijse’s model predicts PVBt values on the order of 10^4^. The adjustment proposed by Schwalbert et al.^26^ successfully reduced these values to the order of 10^2^ and introduces a plateau behavior; however, a discrepancy with the experimental results remains. In our experiments, PVBt* values stayed below 2, indicating that both models fail to capture the correct behavior at low flow rates. Instead of an unbounded increase, the experimental curve approaches a limiting value likely governed by stoichiometric constraints.

The results for partial PVBt* are shown in Figure 5. The green triangle points represent values from experiments conducted at 0.1 mL/min for 0.54, 0.72, and 0.82 PV_injected_, while the pink square points correspond to results at 0.02 mL/min for 0.77, 0.8, and 1.15 PV_injected_. Additionally, the blue circle points represent samples tested at 0.02 mL/min for around 0.8 PV_injected_ under different temperatures, compared to the pink square point for the same duration at 45 °C.

**Figure 5:**
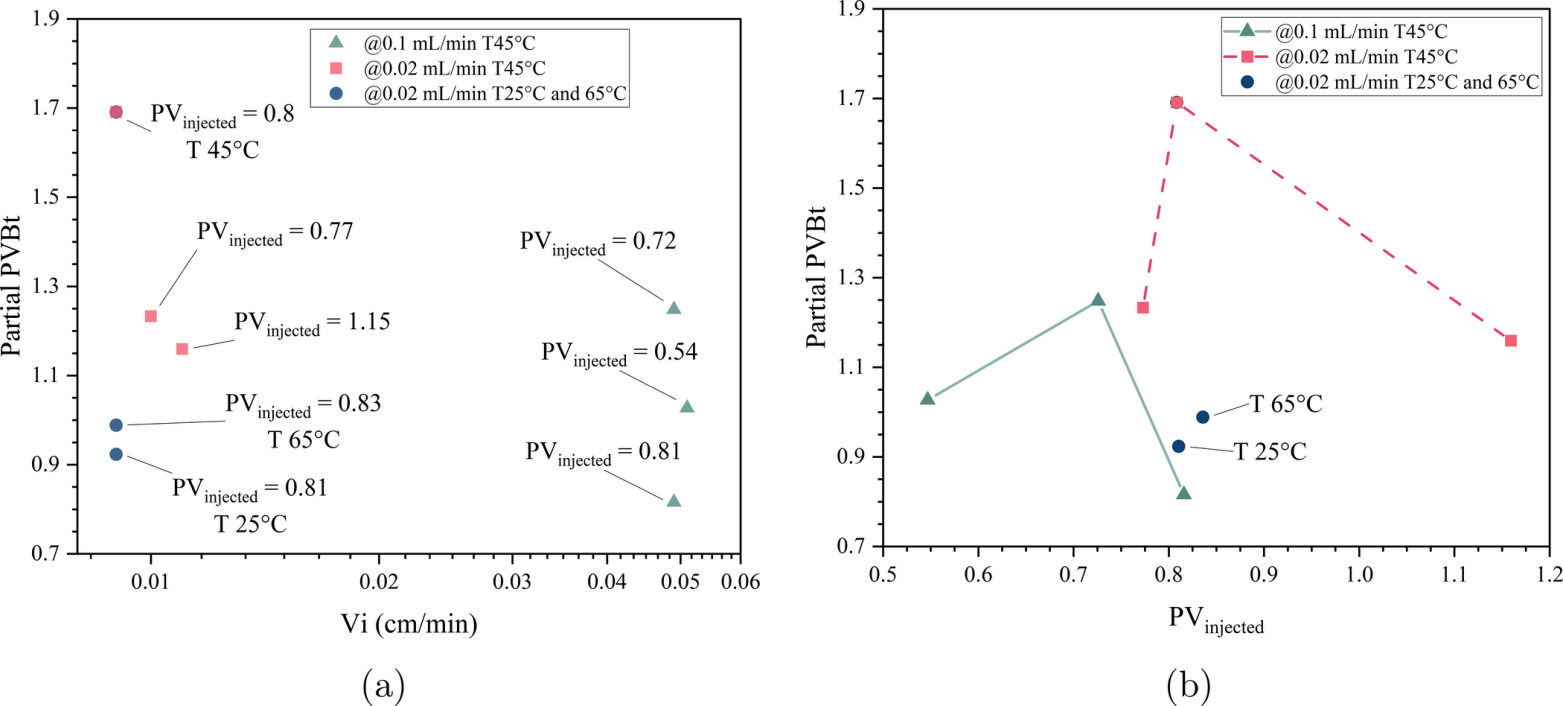
(a) Partial PVBt* at low velocity with 15 wt% HCl in Indiana Limestone in relation to interstitial velocity (cm/min). (b) Partial PVBt* at low velocity in relation to pore volume of acid injected.

The partial PVBt* values increase with injection time at both flow rates. For the 0.1 mL/min, there was an increase of 0.22 in PVBt*, corresponding to a 21.5% rise with an additional 30 min of acid injection. At the lower flow rate of 0.02 mL/min, PVBt* increased by 0.45, a 37.1% rise, with two more hours of injection. The additional acid volumes injected were 3 mL for the 0.1 mL/min flow rate and 2.4 mL for the 0.02 mL/min. However, a more significant increase in PVBt* was observed at the lower flow rate due to the longer injection time and the dominant reaction processes at these conditions. Looking at Figure 5b, the PVBt* values at 0.8 PV_injected_ are very similar, even for different flow rates. This behavior is not observed when examining the effect of temperature at values close to PV_injected_.

At 0.8 PV_injected_ and 0.02 mL/min, varying the temperature produced distinct effects on PVBt. An increase of 0.76 was observed when the temperature was raised from 25 to 45 °C, corresponding to an 83.1% increase. When the temperature was further increased to 65 °C, however, the PVBt* decreased to 0.98. The increase in temperature enhances the acid–rock reaction due to greater molecular agitation, resulting in more extensive dissolution. Under these conditions, an initial increase in PVBt* was followed by a reduction, differing from previously reported trends. This behavior may be influenced by variations in the pore structure and petrophysical properties of the samples, suggesting that additional experiments are required for confirmation. Nevertheless, these results reinforce that, under the evaluated conditions, PVBt values for stronger acids in Indiana Limestone remain on the order of unity.

### Micro-CT Visualization of Wormhole Structure

Figure 6 shows the visualization of wormholes formed in the experiments carried out at low flow rates, with the wormholes structures represented in blue. Each rock sample corresponds to a distinct experiment, with no sample reused at a different flow rate. It is important to note that even cores taken from the same batch of samples may exhibit variations in petrophysical properties, which can lead to some nonlinearities in the results.

**Figure 6:**
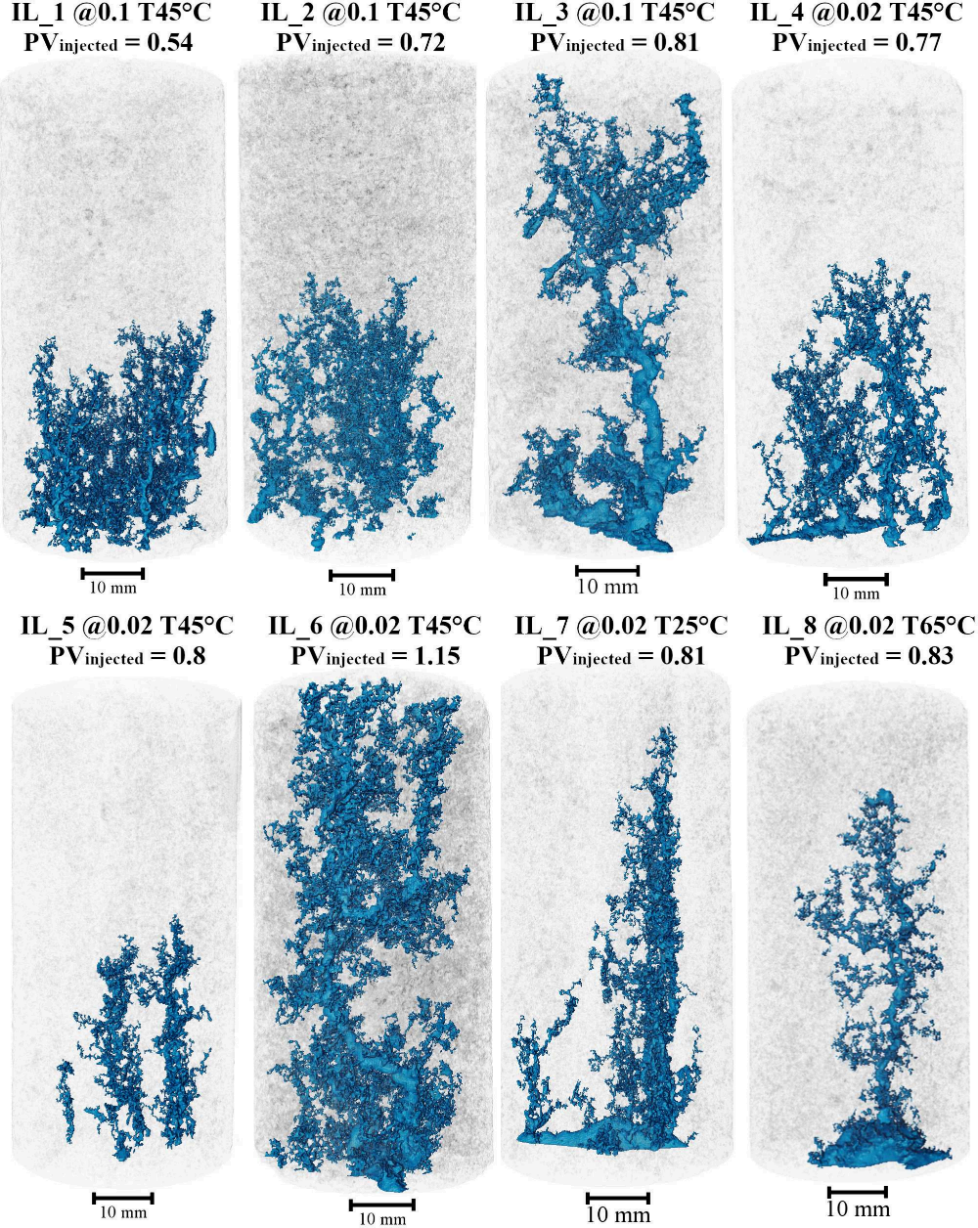
Complete wormhole structure visualization from micro-CT images. The visualization shows the wormhole formed after the experiment, including both partial dissolution results and those that reached breakthrough.

All wormholes presented exhibit several surrounding branches, which is characteristic of this low flow regime. Due to the long duration of the experiments, the acid diffuses through alternative paths, creating branches that are more pronounced in samples IL_1, IL_2 and IL_4 near the inlet. Samples IL_3 and IL_6 were the only ones to reach breakthrough and clearly show a thicker main channel among the branches. This channel was initially identified as the acid propagation after either dissolving the inlet face or generating branches near the inlet and then propagating more efficiently, as observed in experiments with higher PV_injected_.

At higher PV_injected_ values, the inlet branching seen at lower volumes under the same flow rate was not observed, which may reflect sample-to-sample variability rather than systematic differences in the petrophysical properties.

Nevertheless, we note that wormholes are formed even at these low flow rates, and the required PVBt* does not exceed the order of tens for stronger acids, as shown in the PVBt curve from these experiments.

To improve the visualization of the wormhole structure, some branches were removed and the focus was placed on the main dissolution pathway show in Figure 7. One of the first observations is the occurrence of face dissolution in these samples. In some cases, it is more pronounced, such as in IL_7 e IL_8, while in others it is less pronounced but still noticeable, as in IL_2, IL_3, IL_4 and IL_6.

**Figure 7:**
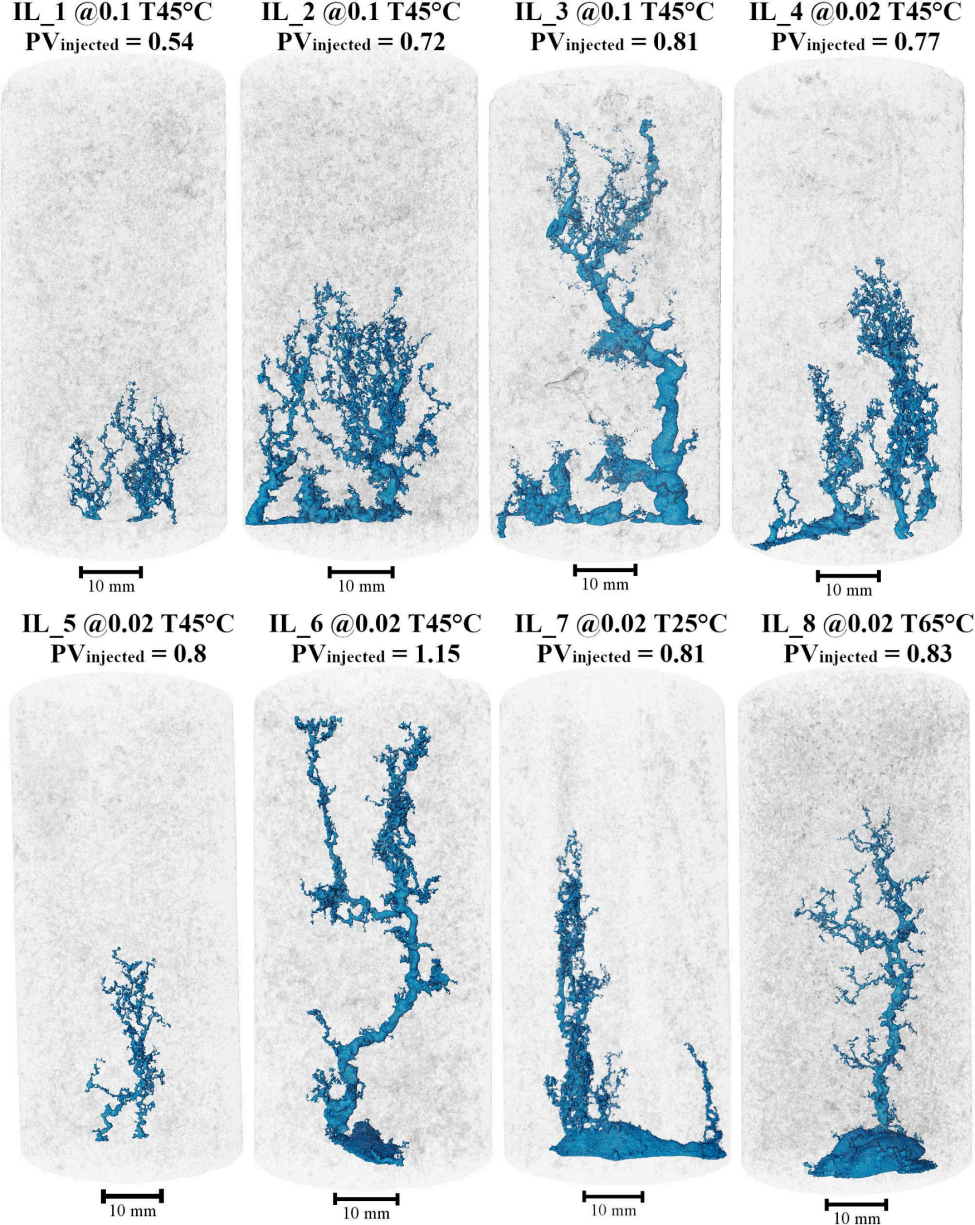
Main wormhole structure visualization from micro-CT images. The wormhole images were processed to highlight only the main channel structures formed after the experiment, both for partial dissolution and for those that reached breakthrough.

At a flow rate of 0.1 mL/min, the first three wormhole images correspond to 0.54, 0.72, and 0.81 PV_injected_. After 0.54 PV_injected_, some channels can be observed propagating toward the center of the sample. With an additional 0.18 PV_injected_ in another experiment, the wormholes extend further into the sample; however, the main dissolution occurs at the entry face, widening the channels. Finally, at 0.81 PV_injected_, face dissolution and the thicker channels are observed, which progressively narrow as they propagate deeper into the sample. Considering the similarities in the structures, in the first stage two channels are formed, and in the following image two main channels are also observed, but with greater thickness and several branches extending up to the midpoint of the sample.

In the third stage, the region near the inlet is similar to the previous stage but with increased thickness; however, the branching observed up to the midpoint of the sample is no longer present. This could be a consequence of variability among the rock samples.

When associating the image results with the partial PVBt* outcome, an increased of 21.5% in PVBt* is observed from 0.54 to 0.72 PV_injected_ due to the limitation in wormhole propagation. However, with a longer injection time, the wormhole fully penetrated the rock, leading to a slightly overestimated of the PVBt. The analysis of the estimated acid consumption corresponds to the visualization of the wormhole formed during the acidizing experiments, where larger PVBt corresponds to a smaller longitudinal area treated with the procedure and less efficient wormhole propagation.

Although these are distinct samples, a similar behavior is observed across experiments. The dynamics of reactivity at low flow rates are particularly intriguing: dissolution initiates and propagates within the porous medium during the first stage, creating channels of similar length without establishing a clear preferential path. Subsequently, longitudinal propagation decreases, and dissolution at the sample inlet increases, leading to the thickening of previously formed channels. The lower acid injection rate causes dynamic variation in acid concentration as it moves through the porous medium. Initially, the spent acid reduces its concentration upon reacting with the rock, diminishing its dissolution power and impeding further propagation. The fresh acid then reacts with the walls of the already formed channels, widening them while further dissolving the rock face due to limited transport during the experiment. After this initial interaction, an acceleration in reactive acid transport is observed, as seen in the third image, allowing the wormhole to propagate to the end.

At a flow rate of 0.02 mL/min, after 0.77 PV_injected_, the formation of two channels is observed, extending beyond the midpoint of the sample, with branching around them and face dissolution near the smaller channel. At 0.8 PV_injected_, some channels are formed, but their morphology is quite different from the previous stage. This behavior may be associated with the distinct pore structure of the sample. With 1.15 PV_injected_, a thicker channel forms, splitting into two near the outlet, and more extensive face dissolution is observed compared to the first stage experiment.

Comparing these wormhole images with the PVBt* values at a flow rate of 0.02 mL/min, from the first to the second stage (0.77 to 0.8 PV_injected_), there is an increase of 37.1% in PVBt*. Although a similar increase in PVBt* is also observed at the other flow rate, the wormhole structures reveal a different dissolution dynamics. At the 0.1 flow rate, face dissolution increased instead of propagating longitudinally through the sample, whereas at the 0.02 flow rate, acid consumption was not as efficient as in the first stage.

The wormhole structure obtained after dissolution experiments at different temperatures at a flow rate of 0.02 mL/min over 10 h show overall similarity, with only minor variations. At both 25 and 65 °C, face dissolution is observed, followed by the propagation of a thicker channel with surrounding branching. At the lower temperature, a second channel forms but does not extend as far, whereas at the higher temperature, more extensive face dissolution and increased branching occur. Higher temperatures enhance molecular agitation and accelerate the reaction rate of the acid with the rock. This change may have concentrated the reaction, increasing face dissolution and forming a thicker channel with more branching around the main channel. These branches tend to spread further due to the difficulty in wormhole propagation under low advective transport and increased reactivity.

A slice located closest to the base of the sample reveals the face dissolution observed in the rock (Figure 8). The rock matrix is shown in gray, while the dissolved regions are represented in black. Most samples exhibit face dissolution, except for IL_1 and IL_5. In the samples where face dissolution occurs, a trend of dissolution through the central region of the sample is observed. This behavior suggests that the initial acid-rock contact does not define a preferential pathway; instead, preferential channels emerge progressively as the dissolution front advances.

**Figure 8:**
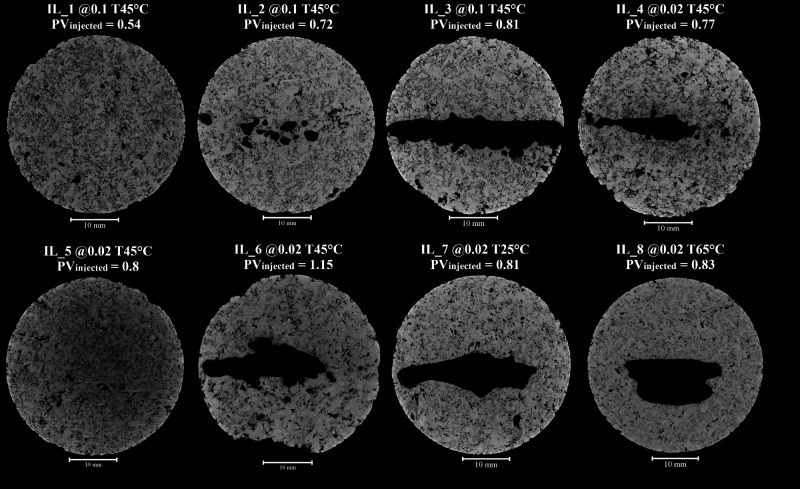
Face dissolution visualization from a slice in micro-CT images. A slice near the inlet was highlighted for each experiment.

At a flow rate of 0.1 mL/min, face dissolution becomes progressively more pronounced with increasing PV_injected_, consistent with previous findings. In the first stage, small channels begin to develop in this region. In the following stage, these channels widen, covering a larger area. Finally, the previously formed channels merge, resulting in significant face dissolution.

This behavior is also observed at a flow rate of 0.02 mL/min, between 0.77 and 1.15 PV_injected_. In the first stage, face dissolution occurs in the middle of the sample, with a few channels forming around it. Subsequently, the face dissolution area increases, accompanied by the formation of additional channels, while some erosion is observed along the rock sides in this section. However, at a 0.8 PV_injected_, no face dissolution is observed. Similar to the wormhole visualization, this result does not follow the same trend observed at 0.1 mL/min.

The more evident dissolution, as shown in the wormhole visualization, is confirmed in the slices for IL_7 and IL_8. At 25 °C, face dissolution is distributed throughout the rock, while at the higher temperature it becomes more concentrated, covering a larger area. These slices are consistent with the 3D wormhole structures presented earlier. When examining all slices at 0.8 PV_injected_, face dissolution is observed in each case, with distinct features but showing significant dissolution overall, except for IL_5.

### Insights on Wormhole Propagation under Low Flow Rates

In this section, the results related to wormhole propagation are analyzed, focusing on its length, velocity, and volume. Figure 9 presents these parameters as a function of the pore volume injected of acid PV_injected_. The green triangles represent measurements obtained at a flow rate of 0.1 mL/min, while the pink squares correspond to experiments at 0.02 mL/min, both conducted at 45 °C. In addition, the blue circles indicate the results from experiments carried out at 25 and 65 °C at the same flow rate of 0.02 mL/min.

**Figure 9:**
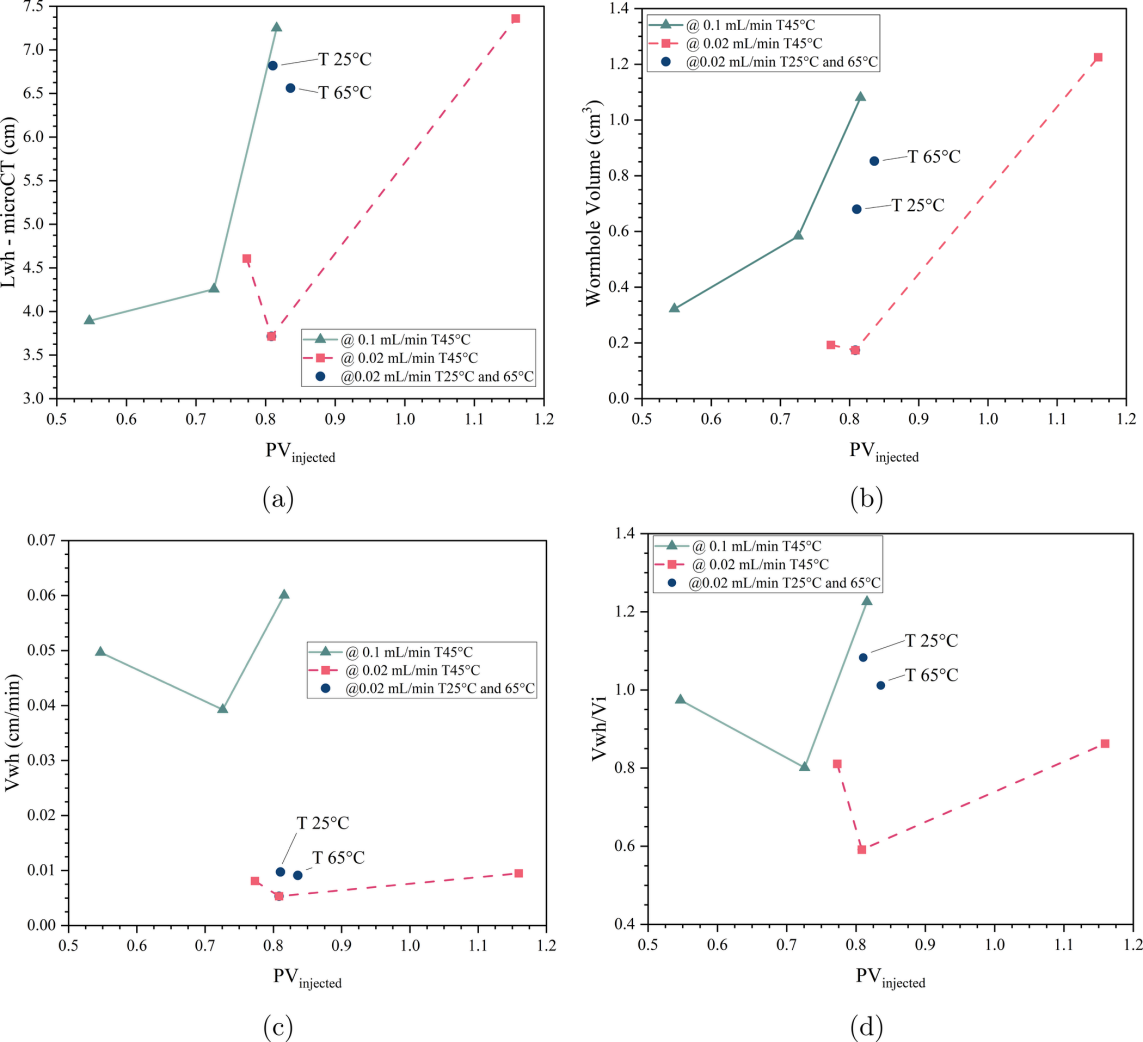
(a) Wormhole length measured through micro-CT in relation to the pore volume of injected acid. (b) Wormhole volume measured through micro-CT in relation to the pore volume of injected acid. (c) Average wormhole propagation velocity at low flow rates in relation to the pore volume of injected acid. (d) Average wormhole propagation normalized by interstitial velocity.

The relationship between wormhole length and pore volume injected of acid follows the previously observed pattern in Figure 9a. For the 0.1 mL/min flow rate, the wormhole initially propagates to a certain length; however, as more acid is injected, further advancement becomes minimal. Eventually, a sudden increase in length is observed in the final stage. This pattern occurs for both flow rates. Initially, propagation occurs through the pores, dissolving the rock and establishing a preferential path. However, the inadequate supply of fresh acid at the wormhole tip hinders further advancement. This results in face enlargement and channel widening until the wormhole is able to propagate more efficiently in the last stage.

Figure 9b illustrates the relationship between wormhole volume and injected acid volume. The wormhole volume follows a similar trend to the wormhole length. A significant portion of the acid volume is consumed in face dissolution, but as fresh acid is replenished along the forming path, the wormhole volume expands until the amount of injected acid is sufficient for breakthrough to occur.

Comparing the wormhole volume values (Figure 9b) with the wormhole length in (Figure 9a), it is evident that at 0.1 mL/min, the wormhole length shows minimal advancement from the first to the second stage, whereas the wormhole volume increases significantly. Between these two stages, the length increases by 0.365 cm (9.3%), while the volume increases by 0.26 *cm* ^3^ (80%). This result supports the discussions about the broadening of the face and the channels formed in the early stages of the reactive process at low flow rates.

Another key observation regarding wormhole length and volume is evident at 0.7 PV_injected_ for both flow rates, 0.1 and 0.02 mL/min. At this stage, both flow rates exhibit similar wormhole lengths; however, the wormhole volume is smaller at the lower flow rate. Examining the wormhole structures in (Figure 6), it is clear that their structures are similar across both flow rates. The main difference is that at the 0.1 mL/min flow rate, a broader spread of dissolution branches is observed, whereas at 0.02 mL/min flow rate, two main propagating channels with branching are observed, but without the lateral spread seen at the other flow rate.

At 0.1 mL/min, thicker channels form at the early stages, followed by more spreading as the wormhole propagates. This behavior suggests that, even at smaller flow rates, the greater velocity allowed advection to play a role, dispersing the acid laterally through the rock while propagating. This facilitated the formation of new paths or the expansion of existing ones during the reaction. In the smaller flow rate, although it shares similar characteristics with the greater flow rate, the reduction in advection allowed the reaction to advance with less spreading compared to the greater flow rate.

The experiments conducted at 25 and 65 °C showed very similar values in Figure 9. These results are consistent with the wormhole images, the base slice, and the PVBt* values. Specifically, IL_7 at 25 °C was more efficient in wormhole propagation, while IL_8 at 65 °C exhibited greater face consumption. However, additional experiments are required to establish a clear trend.

Figure 9c presents the average wormhole propagation velocity at low flow rates, revealing a behavior consistent with the previously discussed results. On the left Figure 9c, the velocity values are shown, while on the right Figure 9d, the values are dimensionless, normalized by the interstitial velocity. The velocity magnitude decreases as the flow rate is reduced. As expected, wormhole propagation velocity is higher at greater flow rates. At both flow rates, an initial velocity is observed, followed by a reduction in velocity during the second stage. Finally, velocity increases in the last stage, surpassing the earlier values.

Despite the efforts to ensure reliability in the experiments, some limitations should be acknowledged. First, the relatively short wormhole propagation observed at partial PVBt may restrict the extent to which wormhole development is captured, potentially influencing breakthrough behavior. Future studies could address this by using longer core samples and allowing higher pore volumes of acid to be injected. Another aspect is the natural heterogeneity of Indiana Limestone, even within samples from the same batch, which introduces variability in pore structure and connectivity and can lead to nonlinearities between experiments. Increasing the number of replicates could help to reduce the impact of these variations. In addition, a sensitivity analysis of the experimental parameters could be valuable, exploring a wider range of conditions, including different acid types, alternative carbonate lithologies, and extended operating parameters.

## Conclusions

This study provides new insights into wormhole propagation behavior in carbonate rocks under low flow rate acidizing conditions using a high-reactivity acid system. A novel experimental methodology was developed to overcome the limitations of traditional PVBt determination at low interstitial velocities, where negligible pressure drop precludes standard breakthrough detection. By implementing time-controlled acid injection and analyzing wormhole propagation through high-resolution micro-CT imaging, it was possible to estimate a partial PVBt (PVBt*) and characterize dissolution behavior at various stages.

The experiments revealed that PVBt* values remained below 2, significantly lower than the predictions from existing semiempirical models, which tend to overestimate breakthrough volumes in this regime. A stabilizing trend was observed in the PVBt curve, suggesting a limiting behavior governed by stoichiometric constraints rather than indefinite growth with decreasing flow rate.

Wormhole propagation followed a consistent three-stage pattern: (1) initial advancement of acid along preferential pore paths, (2) stagnation due to acid depletion and inefficient transport, and (3) renewed propagation upon reestablishment of sufficient acid concentration at the wormhole tip. This behavior was confirmed through measurements of wormhole length, volume, and propagation velocity, as well as micro-CT visualizations.

Flow rate and temperature were found to significantly affect the extent and efficiency of wormhole formation. At lower flow rates, longer injection times resulted in increased PVBt* values and more extensive face dissolution. Increasing the temperature also increases the face dissolution.

Overall, the findings suggest that widely used semiempirical models may require revision to accurately predict wormhole behavior at low flow rates, particularly under operational constraints such as the risk of exceeding the fracturing pressure of the formation. This scenario is common in deep reservoirs as well as in heterogeneous and tight carbonate formations.

Future work should investigate whether this effect also occurs in low-permeability rocks. The experimental methodology and results presented here provide a foundation for refining existing models and support the development of improved acidizing treatment designs for field applications involving constrained flow regimes.
